# Determinants of cesarean delivery: a classification tree analysis

**DOI:** 10.1186/1471-2393-14-215

**Published:** 2014-06-28

**Authors:** Elisa Stivanello, Paola Rucci, Jacopo Lenzi, Maria Pia Fantini

**Affiliations:** 1 Department of Biomedical and Neuromotor Sciences, Alma Mater Studiorum – University of Bologna, Via San Giacomo 12, 40126 Bologna, Italy

**Keywords:** Cesarean delivery, Classification tree analyses, Italy, Determinants

## Abstract

**Background:**

Cesarean delivery (CD) rates are rising in many parts of the world. To define strategies to reduce them, it is important to identify their clinical and organizational determinants. The objective of this cross-sectional study is to identify sub-types of women at higher risk of CD using demographic, clinical and organizational variables.

**Methods:**

All hospital discharge records of women who delivered between 2005 and mid-2010 in the Emilia-Romagna Region of Italy were retrieved and linked with birth certificates. Sociodemographic and clinical information was retrieved from the two data sources. Organizational variables included activity volume (number of births per year), hospital type, and hour and day of delivery. A classification tree analysis was used to identify the variables and the combinations of variables that best discriminated cesarean from vaginal delivery.

**Results:**

The classification tree analysis indicated that the most important variables discriminating the sub-groups of women at different risk of cesarean section were: previous cesarean, mal-position/mal-presentation, fetal distress, and abruptio placentae or placenta previa or ante-partum hemorrhage. These variables account for more than 60% of all cesarean deliveries. A sensitivity analysis identified multiparity and fetal weight as additional discriminatory variables.

**Conclusions:**

Clinical variables are important predictors of CD. To reduce the CD rate, audit activities should examine in more detail the clinical conditions for which the need of CD is questionable or inappropriate.

## Background

Cesarean delivery (CD) rates have increased worldwide during the last decades, especially in middle- and high-income countries [1,2]. CD has become the most common major surgical procedure in many parts of the world, with approximately 18.5 million CDs performed annually [3].

CD was introduced in clinical practice as a life-saving procedure for both the mother and the baby [3]. Several ecological studies have shown an inverse association between CD rates and maternal and infant mortality in low-income countries, where large sectors of the population have limited access to basic obstetric care [2,4]. On the other hand, above a certain level, CD rates do not show an additional benefit for the mother or the baby, and some studies have reported that high CD rates might be linked to negative consequences for maternal and child health [1,2,4-6].

The determinants of CD are very complex and include not only clinical indications, but also economic and organizational factors, the physicians’ attitudes toward birth management, and the social and cultural attitudes of women. Most clinical indications are not absolute and many are very subjective and culture-bound, so there is significant variability among hospitals and countries with respect to CD rates for particular medical indications [7].

Knowledge of CD determinants is a first step in the effort to reduce unnecessary CDs. Italy has one of the highest CD rates in the world, so we conducted a study in a region of Italy with a CD rate of about 30%, with the aim of identifying what combinations of demographic, clinical, and organizational variables best predict which women have a higher risk of CD.

## Methods

Since 1995 in Emilia-Romagna, a northern Italian region with 4.4 million inhabitants, all hospital discharge records (HDRs) have been electronically recorded, using the Hospital Information System. This system includes information about the demographic characteristics of the patient, and diagnoses and procedures during the hospitalization, coded using the International Classification of Diseases, 9th Revision, Clinical Modification (ICD-9-CM).

Moreover, since 2002 the Emilia-Romagna Region has adopted the Certificate of Birth Attendance (CedAP). This registry collects information on sociodemographic characteristics of the parents, obstetric history, prenatal care, and information about the delivery and the newborn.

The HDRs of all women who delivered in the 36 maternity units in the region from 1 January 2005 to 30 June 2010 were extracted and identified using the diagnosis-related group codes 370–375, or the principal or secondary diagnostic codes, V27.xx or 640.xy–676.xy, where y = 1 or 2, or the intervention codes 72.x, 73.2x, 73.5x, 73.6, 73.8, 73.9x, 74.0, 74.1, 74.2, 74.4, and 74.99. A detailed list of codes included in the analysis is provided as Additional file 1. HDRs were linked with the CedAP using the mother's discharge identification code and the year of hospitalization. Linkage was successful in 95% of cases. Data used for the present study include linked records of live births. In case of multiple pregnancy, only one CedAP was retained.

All mothers discharged from a hospital without an operating theater were excluded. Moreover, mothers with one of the following discharge diagnoses were excluded: 656.4 (intrauterine death), V27.1 (single stillborn), V27.4 (twins, both stillborn), and V27.7 (multiple birth, all stillborn).

A CD was identified by diagnosis-related group codes 370 and 371, or ICD-9-CM diagnosis code 669.7, or intervention codes 74.0, 74.1, 74.2, 74.4, and 74.99.

No data were retrieved about past hospitalizations because a previous study [8] indicated that information on comorbidities in the 2 years before delivery did not improve the performance of predictive models of CD.

The following sociodemographic variables were collected: maternal age (<18, 18–24, 25–29, 30–34, 35–39, >39), educational level (university, high, secondary and primary school or less) of the mother and the father, citizenship (Italian, developing countries, non-developing countries other than Italy), and marital status (married, divorced/separated, single, widow, unknown).

The following maternal and fetal clinical factors were retrieved: HIV, diabetes, hypertension, thyroid diseases, lung diseases, other severe comorbidities, genital herpes, substance abuse, eclampsia or pre-eclampsia, abruptio placentae or placenta previa or ante-partum hemorrhage, cephalopelvic disproportion, Rh-isoimmunization, polyhydramnios, oligohydramnios, premature rupture of membranes, cord prolapse, infections of the amniotic cavity, mal-position or mal-presentation, intrauterine growth retardation, dystocia and fetal distress, fetal anomalies, gestational age (pregnancy at term, preterm and post-term), infant birth weight (≤1500, 1501–2499, 2500–3999, ≥4000 g), previous still birth/abortion, previous CD, and multiparity. These factors were defined using the primary and all secondary HDR diagnoses, and/or using CedAP variables. In addition, information on the following organizational variables was retrieved: time of delivery (between 7:01 a.m. and 6:59 p.m., or between 7 p.m. and 7 a.m.), day of delivery (working and non-working days, such as Saturday, Sunday, national holidays), affiliation (teaching or non-teaching hospital) and number of deliveries (i.e., mean number of annual deliveries categorized as: ≤500, 501–799, 800–999, 1000–2499, and ≥2500 deliveries per year, using the classification of the Italian Ministry of Health – SNLG, *Sistema Nazionale Linee Guida*, 2012).

We did not include information about epidural analgesia because this variable only started being collected in 2007. Information on operative vaginal deliveries was available but we did not use it in the statistical analysis because these procedures were rarely used and were uncorrelated or weakly correlated with the CD rate.

### Statistical analysis

The frequencies of all potential determinants of CD rate were calculated. Classification and regression tree analysis (CRT) was used to determine the ability of sociodemographic, clinical and organizational characteristics to discriminate sub-groups of patients with a differential risk of CD. In contrast to traditional statistical models, this non-parametric analysis simultaneously examines interactions between continuous or categorical variables to create a decision tree that does not rely on assumptions about linear relationships between dependent and independent variables. Although this statistical technique has been applied in different medical fields [9,10], to date it has not been used to predict CD. Classification tree analysis is represented graphically as an inverted tree. Beginning with a root node, which includes all cases, the tree branches and grows iteratively by identifying optimal cut points for key discriminating variables in the predictor set. The best discriminating predictor is selected first, and then subsequent predictors are entered into the procedure if they contribute significantly to sub-typing cases that are homogeneous groups in terms of the value of the dependent variable. The final nodes (the “leaves” of the tree) are in fact homogeneous, “pure” nodes, which include all cases with the same value of the dependent variable. The homogeneity of each node was measured using the Gini index. Model over-fitting was avoided by “pruning” the tree: the tree was grown until stopping criteria were met, and then it was trimmed automatically to the smallest sub-tree based on a pre-specified maximum difference in risk.

Goodness-of-fit of the tree was assessed using split-sample validation, i.e. randomly dividing the data into a training set and a test set (75% training and 25% testing) and running the CRT procedure on both sub-samples. If results are comparable, the CRT model fits the data well.

We also conducted sensitivity analyses. First, we reran the CRT model by omitting fetal distress and dystocia. These two conditions might be reported as ex-post justifications for the performed CD, rather than being based on objective clinical assessment [11,12]. This was done to focus on clinical conditions that are less subject to potential bias. Second, the CRT was replicated after excluding some of the organizational variables (i.e., activity volume and hospital type) that are not attributes of the individuals and therefore might alter the statistical properties of the classification trees.

All analyses were conducted using SPSS version 17.0 (Chicago, IL, USA).

### Ethics statement

The study was conducted in conformity with the regulations on data management of the Regional Health Authority of Emilia-Romagna, and with the Italian law on privacy (Art. 20–21, DL 196/2003) [http://www.garanteprivacy.it/web/guest/home/docweb/-/docweb-display/docweb/1115480], published in the Official Journal no. 190 of August 14, 2004), which explicitly exempts the need for ethical approval for using anonymous data (Preamble #8). Data were encrypted prior to the analysis at the regional statistical office, where each patient was assigned a unique identifier. This identifier eliminates the ability to trace the patient’s identity or other sensitive data. As de-identified administrative data are used routinely for health-care management, no specific written informed consent was needed to use the patient information.

## Results

**Figure 1:**
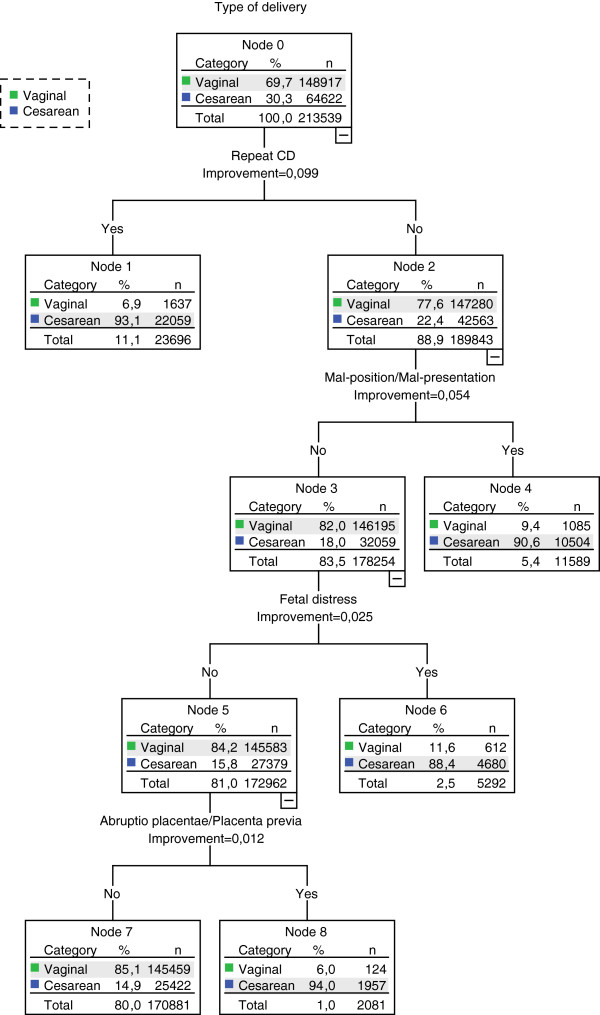
**Classification tree showing sub-groups with different risk of CD (primary analysis).***Note:* This tree includes only those variables that contribute significantly to sub-typing women into homogeneous groups in terms of CD rates (i.e., only relevant predictors of CD). *Abbreviations:CD*, cesarean delivery.

A total of 213,539 women delivered in the Emilia-Romagna Region between 1 January 2005 and 30 June 2010: 148,917 (69.7%) by vaginal deliveries and 64,622 (30,3%) by CDs. Table 1 presents the baseline characteristics of the study population and the CD rate by clinical characteristics. The highest CD rates were observed in women with genital herpes (100.0%), cord prolapse (97.8%), HIV (96.2%), abruptio placentae or placenta previa or ante-partum hemorrhage (95.2%), repeat CD (93.1%), and mal-position/mal-presentation (91.6%).The CRT yielded a segmentation of women into eight sub-groups with different likelihoods of CD. A repeat CD proved to be the key discriminating variable. Among women with a repeat CD (11.1% of the population), no variable proved to be useful to generate further sub-groups. Among women without a repeat CD, mal-presentation characterized a sub-group with a 90.6% probability of CD. In the case of normal presentation, the presence of fetal distress was associated with an 88.4% likelihood of CD. Last, in the absence of fetal distress, abruptio placentae or placenta previa or ante-partum hemorrhage conferred a 94.0% likelihood of CD (Figure 1). The largest sub-group, including 80% of the population without any of the above-mentioned conditions, had a 14.9% risk of CD.

**Table 1:** Number of deliveries and CDs broken down by sociodemographic, clinical and organizational variables in the study population

	**Number of deliveries**	**Number of CDs**	**Percentage of CDs**
**Entire population**	213,539	64,622	30.3
**Age**			
<18	707	113	16.0
18–24	23,677	4,827	20.4
25–29	48,436	12,402	25.6
30–34	76,593	22,921	29.9
35–39	52,265	19,012	36.4
>39	11,861	5,347	45.1
**Citizenship**			
Italian	159,979	50,334	31.5
High-income country	1,997	547	27.4
Low-income country	51,563	13,741	26.7
**Marital status**			
Single	52,995	15,989	30.2
Married	144,153	43,578	30.2
Divorced/separated	5,133	1,904	37.1
Widow	346	141	40.8
Not declared	10,912	3,010	27.6
**Maternal education**			
Primary	9,749	2,749	28.2
Secondary	58,661	18,106	30.9
High-school	96,551	29,118	30.2
University	48,578	14,604	30.1
**Paternal education**			
Primary	6,507	1,898	29.2
Secondary	68,922	20,726	30.1
High-school	81,059	24,468	30.2
University	33,837	10,285	30.4
Unknown	23,214	7,245	31.2
**Comorbidities**			
Thyroid diseases	342	143	41.8
Substance abuse	51	26	51.0
Hypertension	4,198	2,204	52.5
Diabetes	3,105	1,635	52.7
Other severe diseases	1,196	751	62.8
Lung diseases	190	145	76.3
HIV	182	175	96.2
Genital herpes	17	17	100.0
**Obstetrical conditions**			
Premature rupture of membranes	28,971	5,690	19.6
Rh-isoimmunization	1,871	496	26.5
Oligohydramnios	7,550	2,560	33.9
Previous still birth/abortion	36,673	12,665	34.5
Fetal anomalies	2,309	1,076	46.6
Dystocia	7,054	3,583	50.8
Intrauterine growth retardation	6,602	3,695	56.0
Polyhydramnios	491	315	64.2
Infections of the amniotic cavity	176	120	68.2
Eclampsia/pre-eclampsia	3,175	2,319	73.0
Fetopelvic disproportion	2,514	1,843	73.3
Abortion threads/assisted fecundation/supervision of high risk pregnancy	668	459	68.7
Multiple pregnancy	3,222	2,826	87.7
Fetal distress	5,721	5,086	88.9
Mal-position/Mal-presentation of fetus	12,965	11,870	91.6
Repeat CD	23,696	22,059	93.1
Abruptio placentae/placenta previa/ante-partum hemorrhage	2,675	2,546	95.2
Cord prolapse	138	135	97.8
**Pregnancy length**			
At term	193,893	54,205	28.0
Pre-term	15,664	9,430	60.2
Post-term	3,733	910	24.4
Unknown	249	77	30.9
**Infant birth weight (g)**			
≤1500	10,633	6,534	61.5
1501–2499	1,783	1,506	84.5
2500–3999	185,432	52,306	28.2
≥4000	15,471	4,178	27.0
Unknown	220	98	44.5
**Multiparity**			
Nulliparous	117,095	35,700	30.5
Multiparous	96,444	28,922	30.0
**Type of hospital**			
Teaching	58,600	19,868	33.9
Non teaching	154,939	44,754	28.9
**Activity volume (mean number of annual deliveries)**			
100–500	7,286	2,674	36.7
501–799	21,872	6,228	28.5
800–999	19,439	5,070	26.1
1000–2499	96,340	29,255	30.4
≥2500	68,602	21,395	31.2
**Day of delivery**			
Working days	158,265	53,282	33.7
Non-working days	55,274	11,340	20.5
**Time of delivery**			
Daytime (7:01 a.m. – 6:59 p.m.)	142,929	51,333	35.9
Nighttime (7:00 p.m. – 7:00 a.m.)	70,610	13,289	18.8

**Figure 2:**
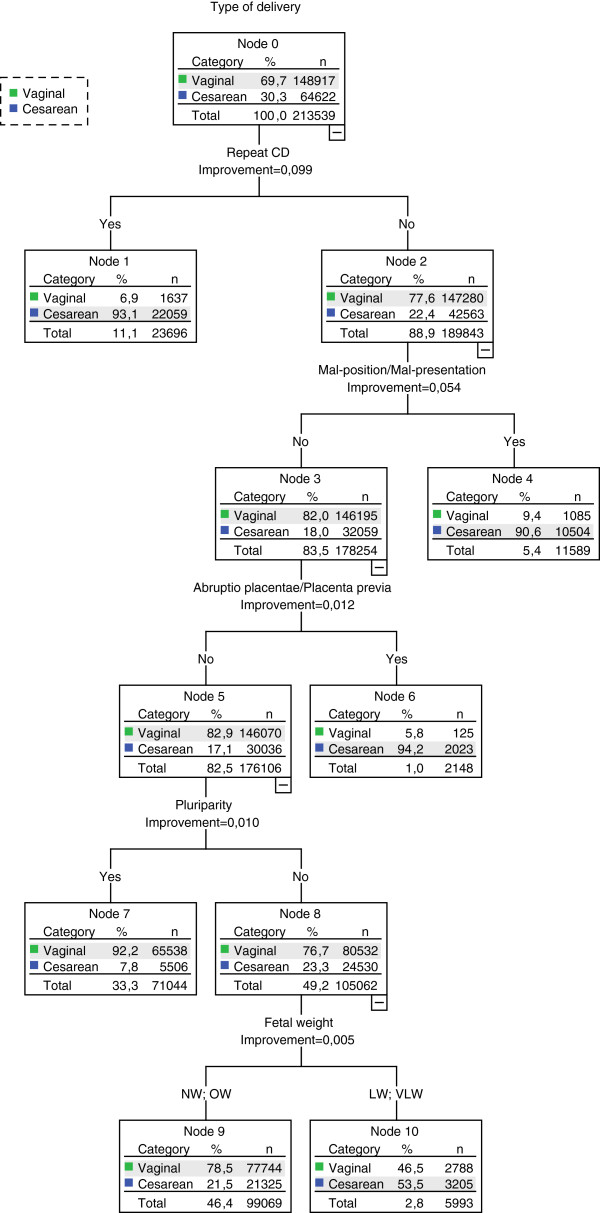
**Classification tree showing sub-groups with different risk of CD (sensitivity analysis).***Note:* This tree includes only those variables that contribute significantly to sub-typing women into homogeneous groups in terms of CD rates (i.e., only relevant predictors of CD). *Abbreviations:CD* cesarean delivery, *NW* normal weight, *OW* overweight, *LW* low weight, *VLW* very low weight.

In summary, the combination of four variables allowed the identification of five mutually exclusive sub-groups (the so-called final nodes of the tree). The CRT model correctly classified 86.5% of deliveries (60.7% of CDs and 97.7% of vaginal deliveries). Moreover, the results of split-sample validation (see Additional file 2) showed that the CRT model optimally fits the data, supporting its external validity.The sensitivity analyses excluding fetal distress and dystocia yielded some differences in the variables discriminating the sub-groups at increased risk of CD. As in the primary analysis, repeat CD and fetal presentation proved to be the most important discriminating variables, followed by abruptio placentae or placenta previa or ante-partum hemorrhage. Fetal weight and parity emerged as new determinants of CD in women without these risk factors (Figure 2). Specifically, in women with single parity, low/very low birth weight was associated with a CD risk of 53.5%. The removal of fetal distress and dystocia generated a model with slightly worse performance compared with the primary model (84.8% of deliveries, 58.5% of CDs and 96.2% of vaginal deliveries correctly classified).

None of the organizational variables proved to be a significant predictor of CD in the CRT models. A sensitivity analysis excluding activity volume and type of hospital yielded results that were identical to those obtained in the primary analysis (data not shown).

## Discussion

The present study sought to identify what combinations of demographic and/or clinical and organizational variables best predicted which women have a higher risk of CD. We correctly identified more than 60.7% of CDs and 97.7% of vaginal deliveries using population-based data on more than 210,000 deliveries, and a CRT model including the presence or absence of repeat CD, mal-presentation, fetal distress, and abruptio placentae or placenta previa or ante-partum hemorrhage. These figures can be interpreted as the positive and negative predictive values of the model, and denote a moderate ability to predict CD and an excellent ability to rule it out.

The sensitivity analysis revealed that fetal weight and multiparity are also important variables. The resulting CRT model had a positive predictive value of 58.5% and a negative predictive value of 96.2%.

This study adds to scientific knowledge by demonstrating the relevance of a number of clinical characteristics of the mother and the fetus on the decision to perform a CD. Compared with the existing research using risk adjustment models, our analytical strategy using classification trees has the potential to identify sub-groups at risk of CD that are characterized by combinations of maternal characteristics, obstetric, and organizational variables.

The variables identified in the present paper as CD predictors are consistent with those reported in other studies [13-19]. Three of them (repeat CD, parity, presentation) are included in the Robson’s Ten Group Classification System (TGCS). The TGCS is considered one of the best classification systems for audit activities [20]. The present study identified other predictors of CD that are not included in the TGCS (e.g., fetal distress, abruptio placentae, placenta previa, ante-partum hemorrhage, and fetal weight) that might be useful for audit activities and inter-hospital comparisons [21].

In the classification tree, only variables that contributed significantly to sub-typing women in terms of CD risk entered the model. Other known CD risk factors, such as HIV, cord prolapse, and genital herpes, were too rare to contribute significantly to sub-typing the women. Literature reviews conducted in the early 2000s [22,23] observed that the four major reported justifications for CD were dystocia, fetal distress, breech presentation and repeat CD. The latter replaced small for gestational age or preterm births, which were important CD determinants in the 1980s. Similarly, the National Sentinel Cesarean Section audit report showed that in England and Wales, the most frequently reported primary indications for cesarean section were presumed fetal distress (22.0% of CDs), failure to progress during labor, i.e. dystocia (20.4%), previous cesarean section (13.8%), and breech presentation (10.8%) [24].

In summary, our study underscores the importance of repeat cesarean section as a CD predictor, and suggests that efforts to reduce CDs should focus on avoiding CDs in primiparous women and on monitoring the appropriateness of CDs in women with previous CDs.

In fact, none of the identified variables was an absolute predictor of CD, as none of them was associated with 100% of CDs. It is not possible to determine how many of these clinically indicated operations were really necessary. Other authors [25] reported similar difficulties in establishing the appropriateness of CD. Sakala [26] argued that the majority of cesareans performed in the United States are attributed to official ‘diagnoses’ that are ambiguous and/or for which a cesarean offers no, or highly questionable, benefit. In particular, the four major indications of CD, previous cesarean, obstructed labor, fetal distress, and breech presentation, are gray areas [27].

None of the organizational variables considered in this study entered the tree because they did not prove to be significant predictors above and beyond the clinical variables. Other organizational factors (or more generally other, non-clinical factors) or women’s preferences [28,29], which were not considered in this analysis, might influence the choice of the type of delivery when clinical indications are present.

The major strength of the present study is that it is a population-based study. However, our results, based on administrative databases, might be affected by lack of accuracy and completeness in coding. In particular, it is possible that omission of ICD codes identifying risk factors is more likely in the group without a CD and that, vice versa, some risk factors are better documented in the group with a CD. This might lead to an information bias. Nevertheless, previous evidence suggests that comorbidities are uncommon among women in reproductive age, who are generally healthy [8].

Although Powell et al. [30] argued that multiple issues regarding the validity of administrative data remain largely unexplored, others [31] suggest that administrative data may be as reliable as data extracted from clinical charts with respect to key outcomes.

Quality improvement is promoted in the Emilia-Romagna Region through training of coders and a regular review of the hospital discharge records database at the Regional Health and Social Care Agency, with feedback to the hospital coders about logical inconsistencies and the presence of systematic errors. The use of ICD-9-CM for coding diagnoses and procedures was established in 2002, thereby facilitating the consistency of coding across operators. Moreover, administrative databases in Emilia-Romagna have proved to have a high degree of completeness and quality [16]. In addition, since some diagnoses (such as fetal distress or placental abnormalities) might be used improperly, we performed sensitivity analyses without these two diagnoses to address this potential bias. A recent study [32] found large differences in the frequency of some types of mal-presentation across hospitals in some Italian regions, which suggests the possibility of improper, or opportunistic, use of this variable as well.

In addition, many organizational risk factors for CD, such as staff type and number, use of procedures, and implementation of audit activities, were not included in the analysis, because this information was not available.

## Conclusions

Our study underscores that the main reasons for performing CDs are clinical. However, some of these ‘clinically’ indicated operations may not be necessary. Therefore, to reduce the CD rate, audit activities should examine in more detail the clinical conditions for which the need of CD is questionable or inappropriate.

## Abbreviations

CD: Cesarean delivery; CedAP: Certificate of Birth Attendance; SNLG: Sistema Nazionale Linee Guida; CRT: Classification and regression tree analysis; HDRs: Hospital discharge records; LW: Low weight; NW: Normal weight; OW: Overweight; VLW: Very low weight.

## Competing interests

The authors declare no competing interests.

## Authors’ contributions

ES conducted part of the analyses, participated in the interpretation of the results and wrote the first draft of the manuscript; PR participated in the design of the study and in the interpretation of the results and helped to draft the manuscript; JL conducted part of the analyses, participated in the interpretation of the results and revised the manuscript; MPF participated in the study design and in the interpretation of the results and revised the manuscript. All authors read and approved the final manuscript.

## Pre-publication history

The pre-publication history for this paper can be accessed here:

http://www.biomedcentral.com/1471-2393/14/215/prepub

## Supplementary Material

Additional file 1 List of diagnosis-related groups, diagnosis and procedure codes included in the study. Click here for file

Additional file 2 **Results of split-sample validation.***Note:* The CRT model was run separately on a training set and a test set (75% and 25% of the study population). Decision trees and classification tables for both sub-samples are provided. *Abbreviations:CD* cesarean delivery, *CRT* classification and regression tree. Click here for file
