# Supplementary material for: Determinants of cesarean delivery: a classification tree analysis
Source: BMC Pregnancy Childbirth. 2014 Jun 28;14:215. doi: 10.1186/1471-2393-14-215 (PMC4090181; doi:10.1186/1471-2393-14-215)
Supplement: Additional file 1 — List of diagnosis-related groups, diagnosis and procedure codes included in the study. [file 1471-2393-14-215-S1.pdf]

LIST OF DIAGNOSIS-RELATED GROUPS, DIAGNOSIS AND PROCEDURE CODES INCLUDED IN THE STUDY

List of diagnosis-related groups

370	Cesarean section with comorbidity or complications
371	Cesarean section without comorbidity or complications
372	Vaginal delivery with complicating diagnoses
373	Vaginal delivery without complicating diagnoses
374	Vaginal delivery with sterilization and/or dilation and curettage
375	Vaginal delivery with operating room procedure except sterilization and/or dilation and curettage

List of ICD-9-CM diagnosis codes

64001	Threatened abortion, delivered, with or without mention of antepartum condition
64081	Other specified hemorrhage in early pregnancy, delivered, with or without mention of antepartum condition
64091	Unspecified hemorrhage in early pregnancy, delivered, with or without mention of antepartum condition
64101	Placenta previa without hemorrhage, delivered, with or without mention of antepartum condition
64111	Hemorrhage from placenta previa, delivered, with or without mention of antepartum condition
64121	Premature separation of placenta, delivered, with or without mention of antepartum condition
64131	Antepartum hemorrhage associated with coagulation defects, delivered, with or without mention of antepartum condition
64181	Other antepartum hemorrhage, delivered, with or without mention of antepartum condition
64191	Unspecified antepartum hemorrhage, delivered, with or without mention of antepartum condition
64201	Benign essential hypertension complicating pregnancy, childbirth, and the puerperium, delivered, with or without mention of antepartum condition
64202	Benign essential hypertension, complicating pregnancy, childbirth, and the puerperium, delivered, with mention of postpartum complication
64211	Hypertension secondary to renal disease, complicating pregnancy, childbirth, and the puerperium, delivered, with or without mention of antepartum condition
64212	Hypertension secondary to renal disease, complicating pregnancy, childbirth, and the puerperium, delivered, with mention of postpartum complication
64221	Other pre-existing hypertension, complicating pregnancy, childbirth, and the puerperium, delivered, with or without mention of antepartum condition
64222	Other pre-existing hypertension, complicating pregnancy, childbirth, and the puerperium, delivered, with mention of postpartum complication
64231	Transient hypertension of pregnancy, delivered , with or without mention of antepartum condition
64232	Transient hypertension of pregnancy, delivered, with mention of postpartum complication
64241	Mild or unspecified pre-eclampsia, delivered, with or without mention of antepartum condition
64242	Mild or unspecified pre-eclampsia, delivered, with mention of postpartum complication
64251	Severe pre-eclampsia, delivered, with or without mention of antepartum condition
64252	Severe pre-eclampsia, delivered, with mention of postpartum complication
64261	Eclampsia, delivered, with or without mention of antepartum condition
64262	Eclampsia, delivered, with mention of postpartum complication
64271	Pre-eclampsia or eclampsia superimposed on pre-existing hypertension, delivered, with or without mention of antepartum condition
64272	Pre-eclampsia or eclampsia superimposed on pre-existing hypertension, delivered, with mention of postpartum complication
64291	Unspecified hypertension complicating pregnancy, childbirth, or the puerperium, delivered, with or without mention of antepartum condition
64292	Unspecified hypertension complicating pregnancy, childbirth, or the puerperium, delivered, with mention of postpartum complication
64301	Mild hyperemesis gravidarum, delivered, with or without mention of antepartum condition
64311	Hyperemesis gravidarum with metabolic disturbance, delivered, with or without mention of antepartum condition

64321	Late vomiting of pregnancy, delivered, with or without mention of antepartum condition
64381	Other vomiting complicating pregnancy, delivered, with or without mention of antepartum condition
64391	Unspecified vomiting of pregnancy, delivered, with or without mention of antepartum condition
64421	Early onset of delivery, delivered, with or without mention of antepartum condition
64511	Post term pregnancy, delivered, with or without mention of antepartum condition
64521	Prolonged pregnancy, delivered, with or without mention of antepartum condition
64601	Papyraceous fetus, delivered, with or without mention of antepartum condition
64611	Edema or excessive weight gain in pregnancy, without mention of hypertension, delivered, with or without mention of antepartum complication
64612	Edema or excessive weight gain in pregnancy, without mention of hypertension, delivered, with mention of postpartum complication
64621	Unspecified renal disease in pregnancy, without mention of hypertension, delivered, with or without mention of antepartum condition
64622	Unspecified renal disease in pregnancy, without mention of hypertension, delivered, with mention of postpartum complication
64631	Recurrent pregnancy loss, delivered, with or without mention of antepartum condition
64641	Peripheral neuritis in pregnancy, delivered, with or without mention of antepartum condition
64642	Peripheral neuritis in pregnancy, delivered, with mention of postpartum complication
64651	Asymptomatic bacteriuria in pregnancy, delivered, with or without mention of antepartum condition
64652	Asymptomatic bacteriuria in pregnancy, delivered, with mention of postpartum complication
64661	Infections of genitourinary tract in pregnancy, delivered, with or without mention of antepartum condition
64662	Infections of genitourinary tract in pregnancy, delivered, with mention of postpartum complication
64671	Liver and biliary tract disorders in pregnancy, delivered, with or without mention of antepartum condition
64681	Other specified complications of pregnancy, delivered, with or without mention of antepartum condition
64682	Other specified complications of pregnancy, delivered, with mention of postpartum complication
64691	Unspecified complication of pregnancy, delivered, with or without mention of antepartum condition
64701	Syphilis of mother, complicating pregnancy, childbirth, or the puerperium, delivered, with or without mention of antepartum condition
64702	Syphilis of mother, complicating pregnancy, childbirth, or the puerperium, delivered, with mention of postpartum complication
64711	Gonorrhea of mother, complicating pregnancy, childbirth, or the puerperium, delivered, with or without mention of antepartum condition
64712	Gonorrhea of mother, complicating pregnancy, childbirth, or the puerperium, delivered, with mention of postpartum complication
64721	Other venereal diseases of mother, complicating pregnancy, childbirth, or the puerperium, delivered, with or without mention of antepartum condition
64722	Other venereal diseases of mother, complicating pregnancy, childbirth, or the puerperium, delivered, with mention of postpartum complication
64731	Tuberculosis of mother, complicating pregnancy, childbirth, or the puerperium, delivered, with or without mention of antepartum condition
64732	Tuberculosis of mother, complicating pregnancy, childbirth, or the puerperium, delivered, with mention of postpartum complication
64741	Malaria in the mother, delivered, with or without mention of antepartum condition
64742	Malaria in the mother, delivered, with mention of postpartum complication
64751	Rubella in the mother, delivered, with or without mention of antepartum condition
64752	Rubella in the mother, delivered, with mention of postpartum complication
64761	Other viral diseases in the mother, delivered, with or without mention of antepartum condition
64762	Other viral diseases in the mother, delivered, with mention of postpartum complication
64781	Other specified infectious and parasitic diseases of mother, delivered, with or without mention of antepartum condition
64782	Other specified infectious and parasitic diseases of mother, delivered, with mention of postpartum complication
64791	Unspecified infection or infestation of mother, delivered, with or without mention of antepartum condition
64792	Unspecified infection or infestation of mother, delivered, with mention of postpartum complication
64801	Diabetes mellitus of mother, complicating pregnancy, childbirth, or the puerperium, delivered, with or without mention of antepartum condition

64802	Diabetes mellitus of mother, complicating pregnancy, childbirth, or the puerperium, delivered, with mention of postpartum complication
64811	Thyroid dysfunction of mother, delivered, with or without mention of antepartum condition
64812	Thyroid dysfunction of mother, delivered, with mention of postpartum complication
64821	Anemia of mother, delivered, with or without mention of antepartum condition
64822	Anemia of mother, delivered, with mention of postpartum complication
64831	Drug dependence of mother, delivered, with or without mention of antepartum condition
64832	Drug dependence of mother, delivered, with mention of postpartum complication
64841	Mental disorders of mother, delivered, with or without mention of antepartum condition
64842	Mental disorders of mother, delivered, with mention of postpartum complication
64851	Congenital cardiovascular disorders of mother, delivered, with or without mention of antepartum condition
64852	Congenital cardiovascular disorders of mother, delivered, with mention of postpartum complication
64861	Other cardiovascular diseases of mother, delivered, with or without mention of antepartum condition
64862	Other cardiovascular diseases of mother, delivered, with mention of postpartum complication
64871	Bone and joint disorders of back, pelvis, and lower limbs of mother, delivered, with or without mention of antepartum condition
64872	Bone and joint disorders of back, pelvis, and lower limbs of mother, delivered, with mention of postpartum complication
64881	Abnormal glucose tolerance of mother, delivered, with or without mention of antepartum condition
64882	Abnormal glucose tolerance of mother, delivered, with mention of postpartum complication
64891	Other current conditions classifiable elsewhere of mother, delivered, with or without mention of antepartum condition
64892	Other current conditions classifiable elsewhere of mother, delivered, with mention of postpartum complication
64901	Tobacco use disorder complicating pregnancy, childbirth, or the puerperium, delivered, with or without mention of antepartum condition
64902	Tobacco use disorder complicating pregnancy, childbirth, or the puerperium, delivered, with mention of postpartum complication
64911	Obesity complicating pregnancy, childbirth, or the puerperium, delivered, with or without mention of antepartum condition
64912	Obesity complicating pregnancy, childbirth, or the puerperium, delivered, with mention of postpartum complication
64921	Bariatric surgery status complicating pregnancy, childbirth, or the puerperium, delivered, with or without mention of antepartum condition
64922	Bariatric surgery status complicating pregnancy, childbirth, or the puerperium, delivered, with mention of postpartum complication
64931	Coagulation defects complicating pregnancy, childbirth, or the puerperium, delivered, with or without mention of antepartum condition
64932	Coagulation defects complicating pregnancy, childbirth, or the puerperium, delivered, with mention of postpartum complication
64941	Epilepsy complicating pregnancy, childbirth, or the puerperium, delivered, with or without mention of antepartum condition
64942	Epilepsy complicating pregnancy, childbirth, or the puerperium, delivered, with mention of postpartum complication
64951	Spotting complicating pregnancy, delivered, with or without mention of antepartum condition
64961	Uterine size date discrepancy, delivered, with or without mention of antepartum condition
64962	Uterine size date discrepancy, delivered, with mention of postpartum complication
64971	Cervical shortening, delivered, with or without mention of antepartum condition
64981	Onset (spontaneous) of labor after 37 completed weeks of gestation but before 39 completed weeks gestation, with delivery by (planned) cesarean section, delivered, with or without mention of antepartum condition
64982	Onset (spontaneous) of labor after 37 completed weeks of gestation but before 39 completed weeks gestation, with delivery by (planned) cesarean section, delivered, with mention of postpartum complication
65101	Twin pregnancy, delivered, with or without mention of antepartum condition
65111	Triplet pregnancy, delivered, with or without mention of antepartum condition
65121	Quadruplet pregnancy, delivered, with or without mention of antepartum condition
65131	Twin pregnancy with fetal loss and retention of one fetus, delivered, with or without mention of antepartum condition

65141	Triplet pregnancy with fetal loss and retention of one or more fetus(es), delivered, with or without mention of antepartum condition
65151	Quadruplet pregnancy with fetal loss and retention of one or more fetus(es), delivered, with or without mention of antepartum condition
65161	Other multiple pregnancy with fetal loss and retention of one or more fetus(es), delivered, with or without mention of antepartum condition
65171	Multiple gestation following (elective) fetal reduction,delivered, with or without mention of antepartum condition
65181	Other specified multiple gestation, delivered, with or without mention of antepartum condition
65191	Unspecified multiple gestation, delivered, with or without mention of antepartum condition
65201	Unstable lie, delivered, with or without mention of antepartum condition
65211	Breech or other malpresentation successfully converted to cephalic presentation, delivered, with or without mention of antepartum condition
65221	Breech presentation without mention of version, delivered, with or without mention of antepartum condition
65231	Transverse or oblique presentation, delivered, with or without mention of antepartum condition
65241	Face or brow presentation, delivered, with or without mention of antepartum condition
65251	High head at term, delivered, with or without mention of antepartum condition
65261	Multiple gestation with malpresentation of one fetus or more, delivered, with or without mention of antepartum condition
65271	Prolapsed arm of fetus, delivered, with or without mention of antepartum condition
65281	Other specified malposition or malpresentation, delivered, with or without mention of antepartum condition
65291	Unspecified malposition or malpresentation, delivered, with or without mention of antepartum condition
65301	Major abnormality of bony pelvis, not further specified, delivered, with or without mention of antepartum condition
65311	Generally contracted pelvis, delivered, with or without mention of antepartum condition
65321	Inlet contraction of pelvis, delivered, with or without mention of antepartum condition
65331	Outlet contraction of pelvis, delivered, with or without mention of antepartum condition
65341	Fetopelvic disproportion, delivered, with or without mention of antepartum condition
65351	Unusually large fetus causing disproportion, delivered, with or without mention of antepartum condition
65361	Hydrocephalic fetus causing disproportion, delivered, with or without mention of antepartum condition
65371	Other fetal abnormality causing disproportion, delivered, with or without mention of antepartum condition
65381	Disproportion of other origin, delivered, with or without mention of antepartum condition
65391	Unspecified disproportion, delivered, with or without mention of antepartum condition
65401	Congenital abnormalities of uterus, delivered, with or without mention of antepartum condition
65402	Congenital abnormalities of uterus, delivered, with mention of postpartum complication
65411	Tumors of body of uterus, delivered, with or without mention of antepartum condition
65412	Tumors of body of uterus, delivered, with mention of postpartum complication
65421	Previous cesarean delivery, delivered, with or without mention of antepartum condition
65431	Retroverted and incarcerated gravid uterus, delivered, with mention of antepartum condition
65432	Retroverted and incarcerated gravid uterus, delivered, with mention of postpartum complication
65441	Other abnormalities in shape or position of gravid uterus and of neighboring structures, delivered, with or without mention of antepartum condition
65442	Other abnormalities in shape or position of gravid uterus and of neighboring structures, delivered, with mention of postpartum complication
65451	Cervical incompetence, delivered, with or without mention of antepartum condition
65452	Cervical incompetence, delivered, with mention of postpartum complication
65461	Other congenital or acquired abnormality of cervix, delivered, with or without mention of antepartum condition
65462	Other congenital or acquired abnormality of cervix, delivered, with mention of postpartum complication
65471	Congenital or acquired abnormality of vagina, delivered, with or without mention of antepartum condition
65472	Congenital or acquired abnormality of vagina, delivered, with mention of postpartum complication

65481	Congenital or acquired abnormality of vulva, delivered, with or without mention of antepartum condition
65482	Congenital or acquired abnormality of vulva, delivered, with mention of postpartum complication
65491	Other and unspecified abnormality of organs and soft tissues of pelvis, delivered, with or without mention of antepartum condition
65492	Other and unspecified abnormality of organs and soft tissues of pelvis, delivered, with mention of postpartum complication
65501	Central nervous system malformation in fetus, delivered, with or without mention of antepartum condition
65511	Chromosomal abnormality in fetus, affecting management of mother, delivered, with or without mention of antepartum condition
65521	Hereditary disease in family possibly affecting fetus, affecting management of mother, delivered, with or without mention of antepartum condition
65531	Suspected damage to fetus from viral disease in the mother, affecting management of mother, delivered, with or without mention of antepartum condition
65541	Suspected damage to fetus from other disease in the mother, affecting management of mother, delivered, with or without mention of antepartum condition
65551	Suspected damage to fetus from drugs, affecting management of mother, delivered, with or without mention of antepartum condition
65561	Suspected damage to fetus from radiation, affecting management of mother, delivered,
65571	Decreased fetal movements, affecting management of mother, delivered, with or without mention of antepartum condition
65581	Other known or suspected fetal abnormality, not elsewhere classified, affecting management of mother, delivered, with or without mention of antepartum condition
65591	Unspecified suspected fetal abnormality, affecting management of mother, delivered, with or without mention of antepartum condition
65601	Fetal-maternal hemorrhage, delivered, with or without mention of antepartum condition
65611	Rhesus isoimmunization, delivered, with or without mention of antepartum condition
65621	Isoimmunization from other and unspecified blood-group incompatibility, delivered, with or without mention of antepartum condition
65631	Fetal distress, affecting management of mother, delivered, with or without mention of antepartum condition
65641	Intrauterine death, affecting management of mother, delivered, with or without mention of antepartum condition
65651	Poor fetal growth, affecting management of mother, delivered, with or without mention of antepartum condition
65661	Excessive fetal growth, affecting management of mother, delivered, with or without mention of antepartum condition
65671	Other placental conditions, affecting management of mother, delivered, with or without mention of antepartum condition
65681	Other specified fetal and placental problems, affecting management of mother, delivered, with or without mention of antepartum condition
65691	Unspecified fetal and placental problem, affecting management of mother, delivered, with or without mention of antepartum condition
65701	Polyhydramnios, delivered, with or without mention of antepartum condition
65801	Oligohydramnios, delivered, with or without mention of antepartum condition
65811	Premature rupture of membranes, delivered, with or without mention of antepartum condition
65821	Delayed delivery after spontaneous or unspecified rupture of membranes, delivered, with or without mention of antepartum condition
65831	Delayed delivery after artificial rupture of membranes, delivered, with or without mention of antepartum condition
65841	Infection of amniotic cavity, delivered, with or without mention of antepartum condition
65881	Other problems associated with amniotic cavity and membranes, delivered, with or without mention of antepartum condition
65891	Unspecified problem associated with amniotic cavity and membranes, delivered, with or without mention of antepartum condition
65901	Failed mechanical induction of labor, delivered, with or without mention of antepartum condition
65911	Failed medical or unspecified induction of labor, delivered, with or without mention of antepartum condition
65921	Maternal pyrexia during labor, unspecified, delivered, with or without mention of antepartum condition
65931	Generalized infection during labor, delivered, with or without mention of antepartum condition
65941	Grand multiparity, delivered, with or without mention of antepartum condition
65951	Elderly primigravida, delivered, with or without mention of antepartum condition
65961	Elderly multigravida, delivered with or without mention of antepartum condition
65971	Abnormality in fetal heart rate or rhythm, delivered, with or without mention of antepartum condition
65981	Other specified indications for care or intervention related to labor and delivery, delivered, with or without mention of antepartum condition

65991	Unspecified indication for care or intervention related to labor and delivery, delivered, with or without mention of antepartum condition
66001	Obstruction caused by malposition of fetus at onset of labor, delivered, with or without mention of antepartum condition
66011	Obstruction by bony pelvis during labor, delivered, with or without mention of antepartum condition
66021	Obstruction by abnormal pelvic soft tissues during labor, delivered, with or without mention of antepartum condition
66031	Deep transverse arrest and persistent occipitoposterior position, delivered, with or without mention of antepartum condition
66041	Shoulder (girdle) dystocia, delivered, with or without mention of antepartum condition
66051	Locked twins, delivered, with or without mention of antepartum condition
66061	Unspecified failed trial of labor, delivered, with or without mention of antepartum condition
66071	Failed forceps or vacuum extractor, unspecified, delivered, with or without mention of antepartum condition
66081	Other causes of obstructed labor, delivered, with or without mention of antepartum condition
66091	Unspecified obstructed labor, delivered, with or without mention of antepartum condition
66101	Primary uterine inertia, delivered, with or without mention of antepartum condition
66111	Secondary uterine inertia, delivered, with or without mention of antepartum condition
66121	Other and unspecified uterine inertia, delivered, with or without mention of antepartum condition
66131	Precipitate labor, delivered, with or without mention of antepartum condition
66141	Hypertonic, incoordinate, or prolonged uterine contractions, delivered, with or without mention of antepartum condition
66191	Unspecified abnormality of labor, delivered, with or without mention of antepartum condition
66201	Prolonged first stage of labor, delivered, with or without mention of antepartum condition
66211	Unspecified prolonged labor, delivered, with or without mention of antepartum condition
66221	Prolonged second stage of labor, delivered, with or without mention of antepartum condition
66231	Delayed delivery of second twin, triplet, etc., delivered, with or without mention of antepartum condition
66301	Prolapse of cord complicating labor and delivery, delivered, with or without mention of antepartum condition
66311	Cord around neck, with compression, complicating labor and delivery, delivered, with or without mention of antepartum condition
66321	Other and unspecified cord entanglement, with compression, complicating labor and delivery, delivered, with or without mention of antepartum condition
66331	Other and unspecified cord entanglement, without mention of compression, complicating labor and delivery, delivered, with or without mention of antepartum condition
66341	Short cord complicating labor and delivery, delivered, with or without mention of antepartum condition
66351	Vasa previa complicating labor and delivery, delivered, with or without mention of antepartum condition
66361	Vascular lesions of cord complicating labor and delivery, delivered, with or without mention of antepartum condition
66381	Other umbilical cord complications complicating labor and delivery, delivered, with or without mention of antepartum condition
66391	Unspecified umbilical cord complication complicating labor and delivery, delivered, with or without mention of antepartum condition
66401	First-degree perineal laceration, delivered, with or without mention of antepartum condition
66411	Second-degree perineal laceration, delivered, with or without mention of antepartum condition
66421	Third-degree perineal laceration, delivered, with or without mention of antepartum condition
66431	Fourth-degree perineal laceration, delivered, with or without mention of antepartum condition
66441	Unspecified perineal laceration, delivered, with or without mention of antepartum condition
66451	Vulvar and perineal hematoma, delivered, with or without mention of antepartum condition
66461	Anal sphincter tear complicating delivery, not associated with third-degree perineal laceration, delivered, with or without mention of antepartum condition
66481	Other specified trauma to perineum and vulva, delivered, with or without mention of antepartum condition
66491	Unspecified trauma to perineum and vulva, delivered, with or without mention of antepartum condition
66501	Rupture of uterus before onset of labor, delivered, with or without mention of antepartum condition
66511	Rupture of uterus during labor, delivered, with or without mention of antepartum condition

66522	Inversion of uterus, delivered, with mention of postpartum complication
66531	Laceration of cervix, delivered, with or without mention of antepartum condition
66541	High vaginal laceration, delivered, with or without mention of antepartum condition
66551	Other injury to pelvic organs, delivered, with or without mention of antepartum condition
66561	Damage to pelvic joints and ligaments, delivered, with or without mention of antepartum condition
66571	Pelvic hematoma, delivered, with or without mention of antepartum condition
66572	Pelvic hematoma, delivered with mention of postpartum complication
66581	Other specified obstetrical trauma, delivered, with or without mention of antepartum condition
66582	Other specified obstetrical trauma, delivered, with mention of postpartum complication
66591	Unspecified obstetrical trauma, delivered, with or without mention of antepartum condition
66592	Unspecified obstetrical trauma, delivered, with mention of postpartum complication
66602	Third-stage postpartum hemorrhage, delivered, with mention of postpartum complication
66612	Other immediate postpartum hemorrhage, delivered, with mention of postpartum complication
66622	Delayed and secondary postpartum hemorrhage, delivered, with mention of postpartum complication
66632	Postpartum coagulation defects, delivered, with mention of postpartum complication
66702	Retained placenta without hemorrhage, delivered, with mention of postpartum complication
66712	Retained portions of placenta or membranes, without hemorrhage, delivered, with mention of postpartum complication
66801	Pulmonary complications of anesthesia or other sedation in labor and delivery, delivered, with or without mention of antepartum condition
66802	Pulmonary complications of anesthesia or other sedation in labor and delivery, delivered, with mention of postpartum complication
66811	Cardiac complications of anesthesia or other sedation in labor and delivery, delivered, with or without mention of antepartum condition
66812	Cardiac complications of anesthesia or other sedation in labor and delivery, delivered, with mention of postpartum complication
66821	Central nervous system complications of anesthesia or other sedation in labor and delivery, delivered, with or without mention of antepartum condition
66822	Central nervous system complications of anesthesia or other sedation in labor and delivery, delivered, with mention of postpartum complication
66881	Other complications of anesthesia or other sedation in labor and delivery, delivered, with or without mention of antepartum condition
66882	Other complications of anesthesia or other sedation in labor and delivery, delivered, with mention of postpartum complication
66891	Unspecified complication of anesthesia and other sedation in labor and delivery, delivered, with or without mention of antepartum condition
66892	Unspecified complication of anesthesia and other sedation in labor and delivery, delivered, with mention of postpartum complication
66901	Maternal distress complicating labor and delivery, delivered, with or without mention of antepartum condition
66902	Maternal distress complicating labor and delivery, delivered, with mention of postpartum complication
66911	Shock during or following labor and delivery, delivered, with or without mention of antepartum condition
66912	Shock during or following labor and delivery, delivered, with mention of postpartum complication
66921	Maternal hypotension syndrome, delivered, with or without mention of antepartum condition
66922	Maternal hypotension syndrome, delivered, with mention of postpartum complication
66932	Acute kidney failure following labor and delivery, delivered, with mention of postpartum complication
66941	Other complications of obstetrical surgery and procedures, delivered, with or without mention of antepartum condition
66942	Other complications of obstetrical surgery and procedures, delivered, with mention of postpartum complication
66951	Forceps or vacuum extractor delivery without mention of indication, delivered, with or without mention of antepartum condition
66961	Breech extraction, without mention of indication, delivered, with or without mention of antepartum condition
66971	Cesarean delivery, without mention of indication, delivered, with or without mention of antepartum condition
66981	Other complications of labor and delivery, delivered, with or without mention of antepartum condition
66982	Other complications of labor and delivery, delivered, with mention of postpartum complication

66991	Unspecified complication of labor and delivery, delivered, with or without mention of antepartum condition
66992	Unspecified complication of labor and delivery, delivered, with mention of postpartum complication
67002	Major puerperal infection, delivered, with mention of postpartum complication
67012	Puerperal endometritis, delivered, with mention of postpartum complication
67022	Puerperal sepsis, delivered, with mention of postpartum complication
67032	Puerperal septic thrombophlebitis, delivered, with mention of postpartum complication
67082	Other major puerperal infection, delivered, with mention of postpartum complication
67101	Varicose veins of legs complicating pregnancy and the puerperium, delivered, with or without mention of antepartum condition
67102	Varicose veins of legs complicating pregnancy and the puerperium, delivered, with mention of postpartum complication
67111	Varicose veins of vulva and perineum complicating pregnancy and the puerperium, delivered, with or without mention of antepartum condition
67112	Varicose veins of vulva and perineum complicating pregnancy and the puerperium, delivered, with mention of postpartum complication
67121	Superficial thrombophlebitis complicating pregnancy and the puerperium, delivered, with or without mention of antepartum condition
67122	Superficial thrombophlebitis complicating pregnancy and the puerperium, delivered, with mention of postpartum complication
67131	Deep phlebothrombosis, antepartum, delivered, with or without mention of antepartum condition
67142	Deep phlebothrombosis, postpartum, delivered, with mention of postpartum complication
67151	Other phlebitis and thrombosis complicating pregnancy and the puerperium, delivered, with or without mention of antepartum condition
67152	Other phlebitis and thrombosis complicating pregnancy and the puerperium, delivered, with mention of postpartum complication
67181	Other venous complications of pregnancy and the puerperium, delivered, with or without mention of antepartum condition
67182	Other venous complications of pregnancy and the puerperium, delivered, with mention of postpartum complication
67191	Unspecified venous complication of pregnancy and the puerperium, delivered, with or without mention of antepartum condition
67192	Unspecified venous complication of pregnancy and the puerperium, delivered, with mention of postpartum complication
67202	Pyrexia of unknown origin during the puerperium, delivered, with mention of postpartum complication
67301	Obstetrical air embolism, delivered, with or without mention of antepartum condition
67302	Obstetrical air embolism, delivered, with mention of postpartum complication
67311	Amniotic fluid embolism, delivered, with or without mention of antepartum condition
67312	Amniotic fluid embolism, delivered, with mention of postpartum complication
67321	Obstetrical blood-clot embolism, delivered, with or without mention of antepartum condition
67322	Obstetrical blood-clot embolism, delivered, with mention of postpartum complication
67331	Obstetrical pyemic and septic embolism, delivered, with or without mention of antepartum condition
67332	Obstetrical pyemic and septic embolism, delivered, with mention of postpartum complication
67381	Other obstetrical pulmonary embolism, delivered, with or without mention of antepartum condition
67382	Other obstetrical pulmonary embolism, delivered, with mention of postpartum complication
67401	Cerebrovascular disorders in the puerperium, delivered, with or without mention of antepartum condition
67402	Cerebrovascular disorders in the puerperium, delivered, with mention of postpartum complication
67412	Disruption of cesarean wound, delivered, with mention of postpartum complication
67422	Disruption of perineal wound, delivered, with mention of postpartum complication
67432	Other complications of obstetrical surgical wounds, delivered, with mention of postpartum complication
67442	Placental polyp, delivered, with mention of postpartum complication
67451	Peripartum cardiomyopathy, delivered, with or without mention of antepartum condition
67452	Peripartum cardiomyopathy, delivered, with mention of postpartum condition
67482	Other complications of puerperium, delivered, with mention of postpartum complication

67492	Unspecified complications of puerperium, delivered, with mention of postpartum complication
67501	Infections of nipple associated with childbirth, delivered, with or without mention of antepartum condition
67502	Infections of nipple associated with childbirth, delivered, with mention of postpartum complication
67511	Abscess of breast associated with childbirth, delivered, with or without mention of antepartum condition
67512	Abscess of breast associated with childbirth, delivered, with mention of postpartum complication
67521	Nonpurulent mastitis associated with childbirth, delivered, with or without mention of antepartum condition
67522	Nonpurulent mastitis associated with childbirth, delivered, with mention of postpartum complication
67581	Other specified infections of the breast and nipple associated with childbirth, delivered, with or without mention of antepartum condition
67582	Other specified infections of the breast and nipple associated with childbirth, delivered, with mention of postpartum complication
67591	Unspecified infection of the breast and nipple associated with childbirth, delivered, with or without mention of antepartum condition
67592	Unspecified infection of the breast and nipple associated with childbirth, delivered, with mention of postpartum complication
67601	Retracted nipple associated with childbirth, delivered, with or without mention of antepartum condition
67602	Retracted nipple associated with childbirth, delivered, with mention of postpartum complication
67611	Cracked nipple associated with childbirth, delivered, with or without mention of antepartum condition
67612	Cracked nipple associated with childbirth, delivered, with mention of postpartum complication
67621	Engorgement of breasts associated with childbirth, delivered, with or without mention of antepartum condition
67622	Engorgement of breasts associated with childbirth, delivered, with mention of postpartum complication
67631	Other and unspecified disorder of breast associated with childbirth, delivered, with or without mention of antepartum condition
67632	Other and unspecified disorder of breast associated with childbirth, delivered, with mention of postpartum complication
67641	Failure of lactation, delivered, with or without mention of antepartum condition
67642	Failure of lactation, delivered, with mention of postpartum complication
67651	Suppressed lactation, delivered, with or without mention of antepartum condition
67652	Suppressed lactation, delivered, with mention of postpartum complication
67661	Galactorrhea associated with childbirth, delivered, with or without mention of antepartum condition
67662	Galactorrhea associated with childbirth, delivered, with mention of postpartum complication
67681	Other disorders of lactation, delivered, with or without mention of antepartum condition
67682	Other disorders of lactation, delivered, with mention of postpartum complication
67691	Unspecified disorder of lactation, delivered, with or without mention of antepartum condition
67692	Unspecified disorder of lactation, delivered, with mention of postpartum complication

List of ICD-9-CM procedure codes

720	Low forceps operation
721	Low forceps operation with episiotomy
7221	Mid forceps operation with episiotomy
7229	Other mid forceps operation
7231	High forceps operation with episiotomy
7239	Other high forceps operation
724	Forceps rotation of fetal head
7251	Partial breech extraction with forceps to aftercoming head
7253	Other partial breech extraction

7254	Total breech extraction with forceps to aftercoming head
726	Forceps application to aftercoming head
7271	Vacuum extraction with episiotomy
7279	Other vacuum extraction
728	Other specified instrumental delivery
729	Unspecified instrumental delivery
7321	Internal and combined version without extraction
7322	Internal and combined version with extraction
7351	Manual rotation of fetal head
7359	Other manually assisted delivery
736	Episiotomy
738	Operations on fetus to facilitate delivery
7391	External version
7392	Replacement of prolapsed umbilical cord
7393	Incision of cervix to assist delivery
7394	Pubiotomy to assist delivery
7399	Other
740	Classical cesarean section
741	Low cervical cesarean section
742	Extraperitoneal cesarean section
744	Cesarean section of other specified type
7499	Other cesarean section of unspecified type
