# Supplementary material for: Determinants of cesarean delivery: a classification tree analysis
Source: BMC Pregnancy Childbirth. 2014 Jun 28;14:215. doi: 10.1186/1471-2393-14-215 (PMC4090181; doi:10.1186/1471-2393-14-215)
Supplement: Additional file 2 — Results of split-sample validation.Note: The CRT model was run separately on a training set and a test set (75% and 25% of the study population). Decision trees and classification tables for both sub-samples are provided. Abbreviations:CD cesarean delivery, CRT classification and regression tree. [file 1471-2393-14-215-S2.pdf]

Training Sample

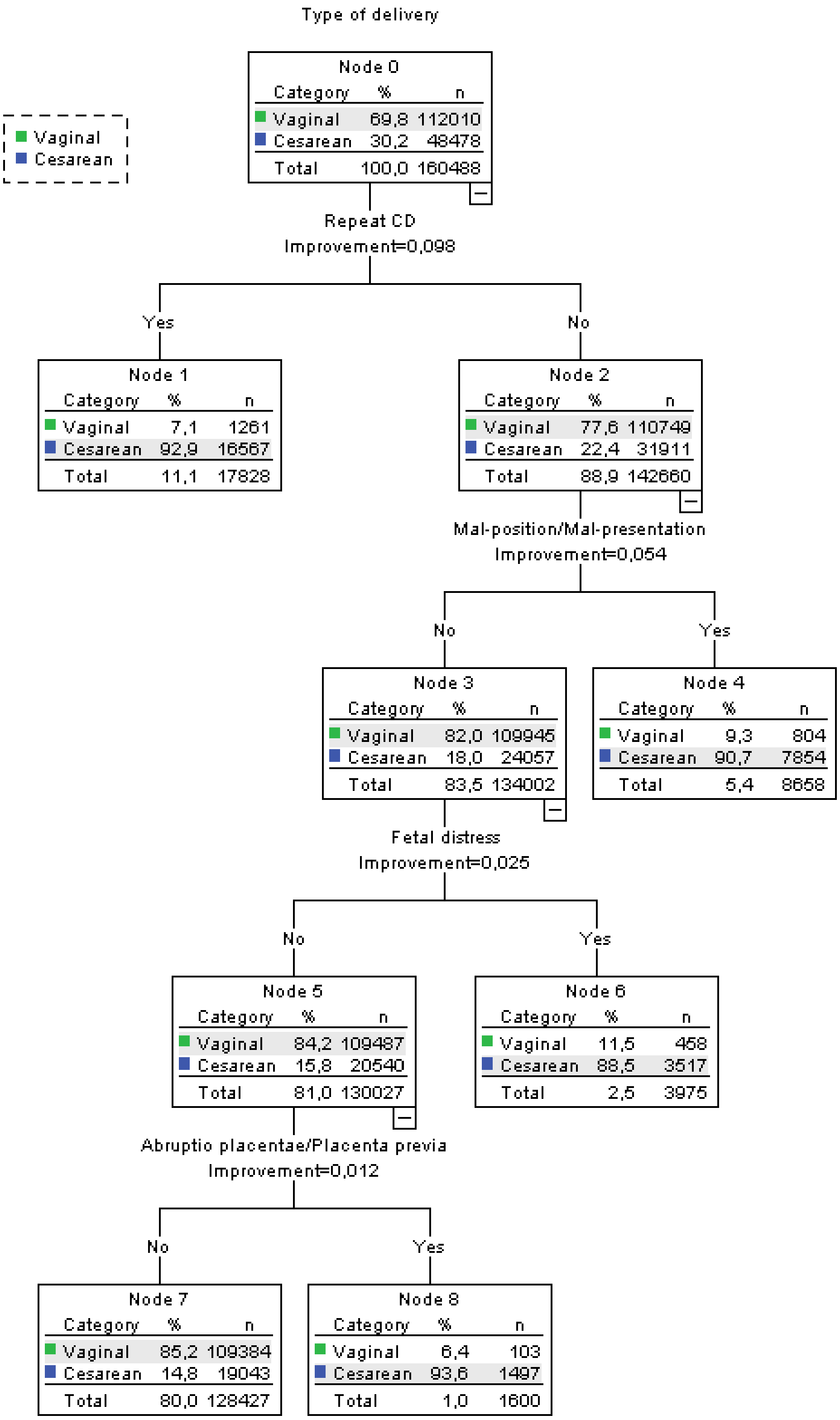

Test Sample

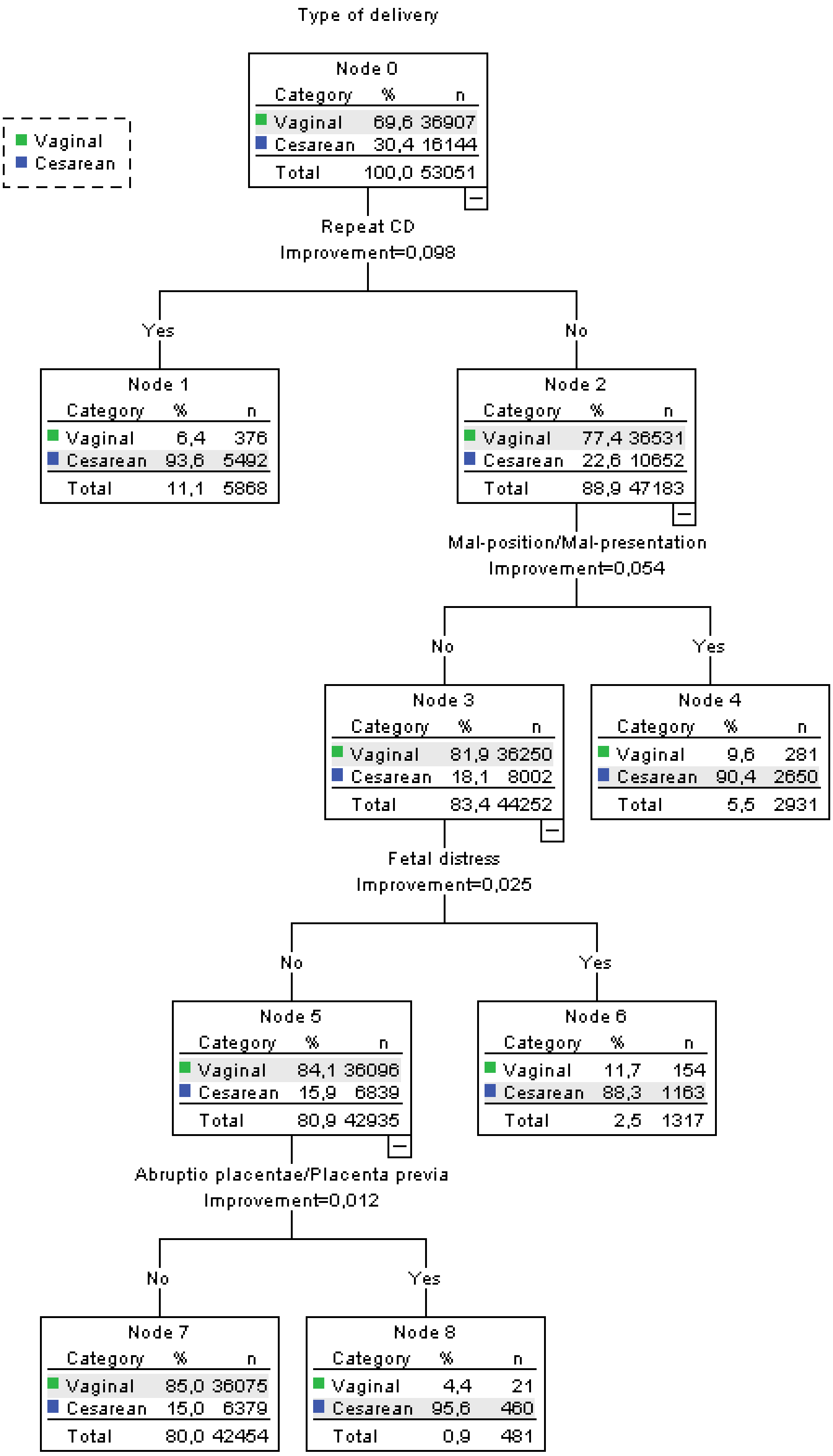

Type of delivery
Vaginal
Cesarean
Node 0
Category % n
Vaginal 69,6 36907
Cesarean 30,4 16144
Total 100,0 53051
Repeat CD
Improvement=0,098
Yes
No
Node 1
Category % n
Vaginal 6,4 376
Cesarean 93,6 5492
Total 11,1 5868
Node 2
Category % n
Vaginal 77,4 36531
Cesarean 22,6 10652
Total 88,9 47183
Mal-position/Mal-presentation
Improvement=0,054
No
Yes
Node 3
Category % n
Vaginal 81,9 36250
Cesarean 18,1 8002
Total 83,4 44252
Node 4
Category % n
Vaginal 9,6 281
Cesarean 90,4 2650
Total 5,5 2931
Fetal distress
Improvement=0,025
No
Yes
Node 5
Category % n
Vaginal 84,1 36096
Cesarean 15,9 6839
Total 80,9 42935
Node 6
Category % n
Vaginal 11,7 154
Cesarean 88,3 1163
Total 2,5 1317
Abruptio placentae/Placenta previa
Improvement=0,012
No
Yes
Node 7
Category % n
Vaginal 85,0 36075
Cesarean 15,0 6379
Total 80,0 42454
Node 8
Category % n
Vaginal 4,4 21
Cesarean 95,6 460
Total 0,9 481

Classification

Sample	Observed	Predicted 0 Vaginal	Predicted 1 Cesarean	Predicted Percent Correct
Training	0 Vaginal	109384	2626	97,7%
	1 Cesarean	19043	29435	60,7%
	Overall Percentage	80,0%	20,0%	86,5%
Test	0 Vaginal	36075	832	97,7%
	1 Cesarean	6379	9765	60,5%
	Overall Percentage	80,0%	20,0%	86,4%

Growing Method: CRT

Dependent Variable: csec_3 Type of delivery
